# Patient Agitation in the Intensive Care Unit: A Concept Analysis

**DOI:** 10.1111/jan.17000

**Published:** 2025-04-25

**Authors:** Anne Mette N. Adams, Diane Chamberlain, Charlotte Brun Thorup, Matthew J. Maiden, Cherie Waite, Hila Ariela Dafny, Kay Bruce, Tiffany Conroy

**Affiliations:** ^1^ College of Nursing and Health Sciences Flinders University Adelaide South Australia Australia; ^2^ Caring Futures Institute Flinders University Adelaide South Australia Australia; ^3^ Research Centre of Health and Applied Technology & Department of Radiography University College Northern Denmark Denmark; ^4^ Department of Critical Care The University of Melbourne Melbourne Victoria Australia; ^5^ Intensive Care Unit Royal Melbourne Hospital Melbourne Victoria Australia; ^6^ Intensive and Critical Care Unit Flinders Medical Centre Adelaide South Australia Australia; ^7^ Southern Adelaide Local Health Network South Australia Australia; ^8^ Central Adelaide Local Health Network Adelaide South Australia Australia

**Keywords:** agitation, concept analysis, critical care, delirium, intensive care unit, nursing, patient and family Centred care, quality and safety

## Abstract

**Aim:**

Exploring the concept of patient agitation in the intensive care unit.

**Background:**

Patient agitation in the intensive care unit is of widespread concern and linked to negative outcomes for patients, staff, and family members. There is currently no consensus on what constitutes agitation in the intensive care context, hindering effective and tailored prevention and management.

**Design:**

Concept Analysis.

**Method:**

Walker and Avant's eight‐step concept analysis approach.

**Data Sources:**

A comprehensive search was carried out in the databases MEDLINE, PsychINFO and CINAHL. A total of 32 papers published between 1992 and 2023 were included, reviewed, and analysed to explore definitions, attributes, antecedents and consequences of patient agitation.

**Results:**

Patient agitation in the intensive care unit is characterised by excessive motor activity, emotional tension, cognitive impairment, and disruption of care, often accompanied by aggression and changes in vital signs. Antecedents encompass critical illness, pharmacological agents and other drugs, physical and emotional discomfort, patient‐specific characteristics and uncaring staff behaviours. Consequences of agitation range from treatment interruptions and poor patient outcomes to the psychological impact on patients, families, and staff.

**Conclusion:**

Agitation in the intensive care unit is a complex issue which significantly impacts patient treatment and clinical outcomes. For healthcare professionals, patient agitation can contribute to high workloads and job dissatisfaction. Due to the complex nature of agitation, clinicians must consider multifaceted strategies and not rely on medication alone. Further research is needed to fully understand patient agitation in the ICU. Such understanding will support the development of improved strategies for preventing and managing the behaviours.

**Implications:**

A clearer understanding of patient agitation supports the development of tailored interventions that improve patient care, guide ICU training, and inform future research.

**Patient or Public Contribution:**

This concept analysis was developed with input from a patient representative.

## 1 Introduction

Patient agitation remains a significant challenge in the intensive care unit (ICU). These behaviours, reported in 32%–70% of ICU patients (Almeida et al. 2016; Burk et al. 2014a; Fraser et al. 2000), often disrupt lifesaving treatment and are linked to a number of adverse events such as line removal (Jaber et al. 2005), unplanned extubation (Chen et al. 2014) and increased length of stay (Woods et al. 2004). Agitation can cause distress not only to the patient experiencing it (Freeman et al. 2022a) but also to the ICU clinicians caring for the patient (Adams et al. 2022) and family members witnessing the episode (Freeman et al. 2022b). Agitation also poses risks to family members and health professionals, leading to both physical harm and emotional exhaustion (Adams et al. 2022; Boehm et al. 2021). While the literature describes different approaches to prevent and manage patient agitation, both pharmacological (Devlin et al. 2018) and nonpharmacological (Adams et al. 2023), successful implementation needs to be based on a clear definition of patient agitation in the ICU. However, there are different understandings of what constitutes agitation, and it is often confused with terms such as delirium, anxiety, and aggression. This confusion complicates the identification, prevention, and management of agitation, highlighting the need for a nuanced, precise definition.

## 2 Background

Despite the importance of prevention and management of patient agitation, one of the barriers to addressing these behaviours lies in the lack of consensus regarding its definition and presentation within the ICU setting. There are existing agitation‐sedation scales, such as the Riker Sedation Agitation Scale (Riker et al. 1999) and the Richmond Agitation Sedation Scale (Sessler et al. 2002) (SAS). However, discrepancies persist in their simplified descriptions of agitation and due to sedation and agitation being on opposite sides of the scales, it can be thought that more sedation is the solution to reducing agitation. Researchers have highlighted multiple limitations to the scales' ability to accurately measure agitation (Adams et al. 2023). Additionally, agitation is frequently confused with terms such as delirium (LeBlanc et al. 2018), anxiety (Tate et al. 2012) and aggression (Saavedra‐Mitjans, Frenette, et al. 2024). Some researchers argue that the term ‘agitation’ should be abandoned altogether due to its lack of nuanced description, potential for moral judgement and implication of ‘danger’ requiring rapid treatment, such as pharmacology or physical restraints, rather than a comprehensive assessment (Fischer et al. 2020). Nevertheless, the word ‘agitation’ remains commonly used in the ICU, where clinicians daily assess levels of sedation and agitation (Doha et al. 2020). Rather than discarding the term, we suggest clinicians gain a better understanding of how agitation is distinguished from other concepts.

Looking outside the ICU, the term ‘agitation’ varies significantly, depending on the exact context in which it is observed. For instance, the International Psychogeriatric Association (Sano et al. 2023) (IPA) limits their definition of agitation to patients with a cognitive disorder. The IPA suggest that emotional distress, as evidenced by excessive motor behaviour or aggression, must be present at least 2 weeks for ‘agitation’ to be ‘diagnosed’. This definition is not applicable to ICU patients who can experience acute agitation and who may or may not be suffering from a cognitive disorder. In dementia care, agitation has been defined as “a response to either internal or external stimuli or both, described by behavioural or verbal disruptiveness, inappropriateness, aggressiveness and repetitiveness, varying in degree depending on older people's individualised thresholds” [(Al Ghassani et al. 2021), 1021]. It is unclear if this definition applies to ICU patients, as not all agitated patients in the ICU are aggressive or exhibit repetitive behaviours.

Without a solid and nuanced understanding of patient agitation in the ICU, clinicians may be tempted to focus less on the causes of agitation and more on urgently treating the symptoms and behaviours, which may not be helpful to the patient in the long term. With no clear understanding of agitation, efforts in identifying strategies for preventing and managing agitation are likely to fall short. Therefore, this paper aims to provide a concept analysis of agitation. The objectives include identifying existing definitions of agitation in the ICU, examining the antecedents, attributes, and consequences of agitation, and presenting illustrative case examples.

## 3 Methods

This concept analysis follows Walker and Avant's (2019) eight‐step guide, which offers a straightforward, pragmatic and recognised approach to concept analysis (Beecher et al. 2019). The guide includes: (1) selecting a topic; (2) determining the aim; (3) identifying uses of the concept; (Jaber et al. 2005) determining defining attributes; (Chen et al. 2014) identifying a model case; (Woods et al. 2004) identifying borderline cases; (Freeman et al. 2022a) identifying antecedents and consequences; and (Adams et al. 2022) defining empirical references of the concept. As suggested by Walker and Avant (2019), the steps were carried out in a dynamic and iterative way, where authors continuously moved back and forth between steps.

### 3.1 Data Sources

A comprehensive search strategy was developed in consultation with a librarian and carried out across the databases MEDLINE, PsychINFO, and CINAHL. The search utilised the keywords ‘agitation’ and ‘intensive care’ and various synonyms of these terms (see Appendix 1).

Papers were considered if they met the following criteria: (Almeida et al. 2016) provided a definition of agitation and/or aimed to explore the concept of agitation, and (Burk et al. 2014a) described the antecedents and/or the consequences and/or the attributes of agitation in the ICU. Eligible papers needed to be peer‐reviewed primary studies (qualitative, quantitative and mixed‐methods), reviews or discussion papers published in English and published before November 2023. Papers focusing on paediatric or neonatal ICUs or other contexts, as well as papers focusing solely on the management of agitation, such as testing interventions for agitation, were excluded.

**FIGURE 1:**
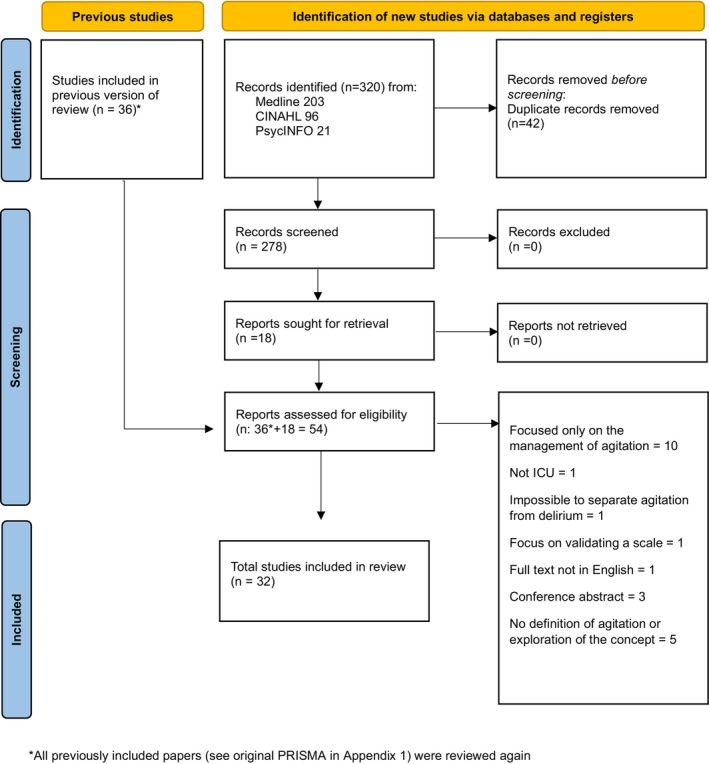
PRISMA (Page et al. 2021) Updated search Nov 2023.

The original search was conducted in August 2022 (see Appendix 2). This was updated in November 2023 (see Figure 1). The original search yielded 36 papers; however, after a thorough review and refinement of the inclusion criteria by the research team, it was determined that only 28 of the original papers met the specified criteria for inclusion. The updated search identified an additional four papers, bringing the total to 32 included studies. A data extraction form was developed and utilised for all studies.

## 4 Findings

### 4.1 Identifying Definitions of Agitation

Table 1 describes how agitation has been defined^1^ in the included literature, noting that 44% (*n* = 14) of studies did not provide a definition of agitation. Instead, these 14 studies described agitation using a scale (Chen et al. 2014; Woods et al. 2004; Burk et al. 2014b; Huang et al. 2018; Lucidarme et al. 2010; Malinowski et al. 2020; Wang et al. 2020), and/or described the attributes of agitation (Heily et al. 2024, 2023; O'Connor et al. 2014; Prendergast et al. 2023; Shapira 2002; Stewart et al. 2019; Williamson et al. 2020).

**TABLE 1:** Definitions of agitation within the ICU literature.

Reference	Definition
Azimaraghi et al. (2023)	Refer to the definition of Chevrolet et al. (Chevrolet and Jolliet 2007) and the definition in the Diagnostic and Statistical Manual of Mental disorders “excessive motor activity associated with a feeling of inner tension. The activity is described as non‐productive and repetitious and consists of behaviours such as pacing, fidgeting, wringing of the hands, pulling of clothes, and inability to sit still” (Azimaraghi et al. 2023, 238).
Adams et al. (2022)	Refer to the definition of Chevrolet et al. (Chevrolet and Jolliet 2007).
Freeman et al. (2022a)^a^	Agitation, a condition of psychomotor disturbance … the patient can show unintentional and purposeless movement stemming from anxiety and accompanied by disorganised thought (Whitehouse et al. 2014)
Freeman et al. (2022b)	Refer to the definition by Whitehouse et al. (Whitehouse et al. 2014)
Aubanel et al. (2020)	Agitation can be defined as an important elevation of motor and psychological activity and may be associated with disorganised thinking (Aubanel et al. 2020, 639).
Freeman et al. (2018)	Refer to the definition by Whitehouse et al. (Whitehouse et al. 2014)
Mahmood et al. (2018)	Agitation is an acute cognitive dysfunction… It is a state of restlessness accompanied with excessive and aimless motor and cognitive activities. It is characterised by changes or fluctuation in baseline mental status, disorganised thinking or an altered level of consciousness (Mahmood et al. 2018, 105).
Almeida et al. (2016)	It is characterised by increased motor and mental activity that manifests as inappropriate behaviour, disorganised thoughts, and a loss of self‐control over actions (Almeida et al. 2016, 413).
Jaber et al. (2005)	Frequent movements of head, arms, or legs, and/or bucking the ventilator that persisted despite attempts of staff to calm the patient (Jaber et al. 2005, 2750)
Burk et al. (2014b)	Agitation is excessive restlessness, or non‐purposeful physical activity thought to be caused or exacerbated by pain, anxiety, endotracheal tube irritation, or other unpleasant events (Burk et al. 2014b, 1).
Whitehouse et al. (2014)	Agitation is a psychomotor disturbance characterised by a marked increase in both motor and psychological activities, often accompanied by a loss of control of action and a disorganisation of thought (Whitehouse et al. 2014, 15).
Tate et al. (2012)	Agitation is defined as “disquietude”, “violent motion,” and “tumultuous emotion” and involves increased intensity in behavioural and psychological dimensions. Agitation is a visible cue that can occur in isolation or accompany extreme anxiety, delirium, or brain dysfunction (Tate et al. 2012, 158).
Honiden and Siegel (2010)	Physical and psychological distress, commonly characterised as a state of excessive motor activity (Honiden and Siegel 2010, 188).
Chevrolet and Jolliet (2007)	Agitation is a psychomotor disturbance characterised by a marked increase in both motor and psychological activities, often accompanied by a loss of control of action and a disorganisation of thought (Chevrolet and Jolliet 2007, 1).
Siegel (2003)	Agitation is characterised by excess motor activity and is driven by internal factors, such as disease, pain, anxiety, and delirium (Siegel 2003, 713).
Cohen et al. (2002)	(1) Violent motion, (2) strong or tumultuous emotion…encompassing both physical and emotional distress (Cohen et al. 2002, 97).…[occurs] along a continuum of continuous changing physiologic states with varying behaviours and responses, affecting each patient differently within the severity and complexity of their condition (Cohen et al. 2002, 101).
Crippen (1999)	The term ‘agitation’ describes a syndrome of excessive motor activity, usually no purposeful and associated with internal tension (Crippen 1999, 35).
Fraser et al. (2000)	Excessive motor activity associated with internal tension (Hansen‐Flaschen 1994, 76).

^a^ In this manuscript, ‘Freeman et al. (2022a)’ and ‘Freeman et al. (2022b)’ refer to two different articles published by the same author in 2022.

Table 1 illustrates how the definition of agitation has changed over time. Early definitions provided broad descriptions acknowledging both a physical and a psychological dimension of agitation (Cohen et al. 2002; Crippen 1999). More recent definitions offer a more holistic perspective, acknowledging multiple internal and external factors contributing to agitation. This change suggests an improved understanding of ICU patient agitation over time. Although there seems to be a broad consensus that agitation consists of both physical (e.g., increased motor activity, restlessness) and psychological components (e.g., disorganised thoughts and emotional distress), definitions in the literature vary in their emphasis between these aspects. This variation among definitions reflects the complex nature of agitation and the lack of unified understanding.

The definition by Chevrolet and Jolliet (2007) was referred to by several authors (Adams et al. 2022; Freeman et al. 2022b, 2018; Azimaraghi et al. 2023) in the ICU literature. The authors (Chevrolet and Jolliet 2007) define agitation as “a psychomotor disturbance characterized by a marked increase in both motor and psychological activities, often accompanied by a loss of control of action and a disorganization of thought” (Whitehouse et al. 2014, p. 15). While the definition provides a good starting point, it does not describe the antecedents and consequences of agitation and there may be ambiguity related to the terms ‘psychomotor disturbance’ and ‘disorganisation of thoughts’. For example, while psychomotor disturbance suggests a disruption in the coordination between mental state and physical actions, it does not specify which actions this entails, for example, fidgeting, thrashing, pulling, restlessness and if these are voluntary or involuntary. Disorganisation of thoughts is a very broad term that could include many symptoms such as confusion, hallucinations, or impaired decision‐making.

### 4.2 Determining the Defining Attributes

Defining attributes, or characteristics, are descriptions of the concepts that appear repeatedly (Walker and Avant 2019). This concept analysis identified six attributes, which are explored below (see Appendix 3 for an overview of the extracted data). The first four, excessive motor activity, emotional tension, interrupting or resisting care and cognitive impairment, were the most mentioned attributes and can perhaps be seen as the core attributes. The last two attributes, including aggressive behaviours and change of vital signs, were mentioned less frequently and may, therefore, not always be present in agitated patients. Each attribute is explored further below.

#### 4.2.1 Excessive Motor Activity

Excessive motor activity is described most frequently in the reviewed literature and includes excessive or a marked increase in motor activities (Almeida et al. 2016; Adams et al. 2022; Prendergast et al. 2023; Chevrolet and Jolliet 2007; Azimaraghi et al. 2023; Whitehouse et al. 2014; Aubanel et al. 2020; Mahmood et al. 2018; Honiden and Siegel 2010; Siegel 2003; Crippen 1999), restlessness (Fraser et al. 2000; Tate et al. 2012; O'Connor et al. 2014; Prendergast et al. 2023; Shapira 2002; Stewart et al. 2019; Williamson et al. 2020; Mahmood et al. 2018; Cohen et al. 2002; Freeman et al. 2018), hyperactivity (Stewart et al. 2019), and repetitive (Williamson et al. 2020; Azimaraghi et al. 2023) or frequent (Fraser et al. 2000; Sessler et al. 2002; Cohen et al. 2002) movements. These behaviours are often uncontrolled or unintentional (Heily et al. 2023) and may lack apparent purpose (Sessler et al. 2002; Heily et al. 2024; Mahmood et al. 2018; Freeman et al. 2018). Excessive motor activity can involve small muscle groups, characterised by fidgeting (Fraser et al. 2000; Jaber et al. 2005; Stewart et al. 2019; Azimaraghi et al. 2023; Cohen et al. 2002) and pulling at dressings or bedsheets (Tate et al. 2012; Shapira 2002; Cohen et al. 2002), or involve large muscle groups, including rhythmic head movements (Tate et al. 2012), shaking (Heily et al. 2024, 2023; Whitehouse et al. 2014), thrashing (Fraser et al. 2000; Riker et al. 1999; Heily et al. 2023; Shapira 2002; Stewart et al. 2019; Cohen et al. 2002; Freeman et al. 2018), kicking (Shapira 2002), rocking (Williamson et al. 2020), pacing (Stewart et al. 2019; Azimaraghi et al. 2023), banging on the side rails (Tate et al. 2012) or attempts to sit up or get out of bed (Fraser et al. 2000; Jaber et al. 2005).

#### 4.2.2 Emotional Tension

Emotional tension refers to a troubled state of mind, inner tension (Stewart et al. 2019; Azimaraghi et al. 2023; Crippen 1999; Freeman et al. 2018), tumultuous emotion (Cohen et al. 2002), an increase in psychological or mental activities (Almeida et al. 2016; Adams et al. 2022; Chevrolet and Jolliet 2007; Whitehouse et al. 2014; Aubanel et al. 2020) and emotional instability, such as rapid and excessive emotional fluctuations (Williamson et al. 2020). This attribute encompasses a range of emotions such as fear (Fraser et al. 2000), anxiety (Burk et al. 2014a; Fraser et al. 2000; Freeman et al. 2022a; Riker et al. 1999; Sessler et al. 2002; Tate et al. 2012; Siegel 2003; Cohen et al. 2002), paranoia (Fraser et al. 2000), panic (Freeman et al. 2022a) psychological distress (Heily et al. 2023; O'Connor et al. 2014; Stewart et al. 2019; Honiden and Siegel 2010) and unease (Burk et al. 2014a; Fraser et al. 2000; Sessler et al. 2002; Tate et al. 2012). Manifestations of emotional tension included grimacing, tensing facial muscles, tensing of the body, moaning, wincing, or shouting (Tate et al. 2012; Shapira 2002; Cohen et al. 2002).

#### 4.2.3 Disruption of Care

Disruption of care involves resisting, removing, or moving away from therapeutic treatment or devices, leading to an increased risk of adverse events and complicating patient care and management. These interruptions compromise the quality of care by hindering the effect of treatment and increasing the likelihood of negative outcomes (Heily et al. 2023; Chevrolet and Jolliet 2007). Patients may try to sit up or get out of bed (Fraser et al. 2000; Riker et al. 2001), attempt to remove catheters, lines and tubes (Fraser et al. 2000; Jaber et al. 2005; Riker et al. 1999; Heily et al. 2023; Shapira 2002; Williamson et al. 2020; Whitehouse et al. 2014; Aubanel et al. 2020; Cohen et al. 2002; Freeman et al. 2018) and pull at dressings, bedsheets (Shapira 2002; Cohen et al. 2002) and physical restraints (Fraser et al. 2000; Tate et al. 2012). Inadequate mechanical ventilation can occur due to patients biting the endotracheal tube (Fraser et al. 2000; Riker et al. 1999) or ‘fighting the ventilator’ causing high pressures and ventilator dyssynchrony (Fraser et al. 2000; Sessler et al. 2002; Williamson et al. 2020; Cohen et al. 2002). Agitated patients often resist treatment (Adams et al. 2022; Tate et al. 2012; Prendergast et al. 2023; Shapira 2002; Stewart et al. 2019), for instance by protesting loudly (Fraser et al. 2000) and becoming uncooperative (Fraser et al. 2000; Shapira 2002; Williamson et al. 2020; Mahmood et al. 2018).

#### 4.2.4 Cognitive Impairment

Cognitive impairment refers to a change in mental status and a diminished ability to think rationally. This attribute makes it challenging for patients to fully understand their environment and communicate their needs. Cognitive impairment manifests through disorganised thinking (Almeida et al. 2016; Freeman et al. 2022a; Chevrolet and Jolliet 2007; Whitehouse et al. 2014; Aubanel et al. 2020; Mahmood et al. 2018), irrational thoughts (Cohen et al. 2002), difficulties concentrating (Cohen et al. 2002), disorientation and confusion (Fraser et al. 2000; Williamson et al. 2020; Cohen et al. 2002) and impaired memory (Cohen et al. 2002). For example, a patient might complain about pain when the real issue is a need to urinate (Cohen et al. 2002). Agitated patients may also experience an altered level of consciousness (Mahmood et al. 2018), hallucinations or delusions (Williamson et al. 2020; Riker et al. 2001). Cognitive impairment can result in inappropriate behaviours (Almeida et al. 2016; Burk et al. 2014a), an inability to listen, communicate and follow commands (Jaber et al. 2005; Williamson et al. 2020; Cohen et al. 2002). While some authors describe a loss of self‐control (Almeida et al. 2016; Chevrolet and Jolliet 2007; Whitehouse et al. 2014), others describe how actions in agitated patients may be intentional and conscious (Shapira 2002).

#### 4.2.5 Aggressive Behaviour

Aggressive behaviour was mentioned less frequently in the literature. The authors described how agitated patients could exhibit violent (Jaber et al. 2005; Sessler et al. 2002; Williamson et al. 2020; Cohen et al. 2002), hostile (O'Connor et al. 2014), angry (Tate et al. 2012; Williamson et al. 2020) aggressive and dangerous (Burk et al. 2014a; Adams et al. 2022; Sessler et al. 2002) behaviours. These patients may become combative and threatening (Fraser et al. 2000; Heily et al. 2024) towards care providers posing a risk to themselves, their family members and healthcare staff.

#### 4.2.6 Changes of Vital Signs

Changes in vital signs can be indicators of agitated behaviours. These changes include elevated heart rate (Tate et al. 2012; Shapira 2002; Cohen et al. 2002) and respiratory rate (Tate et al. 2012; Shapira 2002; Cohen et al. 2002), increased blood pressure (Tate et al. 2012; Cohen et al. 2002) and metabolic rate (Cohen et al. 2002), and increased coughing (Tate et al. 2012). Agitation can also result in autonomic hyperactivity (Stewart et al. 2019) and haemodynamic instability (Heily et al. 2024, 2023).

From this comprehensive analysis, patient agitation in the ICU can be characterised as excessive motor activity, emotional tension, disruption of care and cognitive impairment, often accompanied by aggression and changes in vital signs.

### 4.3 Illustrative Cases

#### 4.3.1 A Model Case

According to Walker and Avant (Walker and Avant 2019) a model case gives the reader an example of the concept and should represent a made‐up or real example of the phenomenon of interest (Walker and Avant 2019). The case in Box 1 is fictional and based on the author's previous experiences as a nurse in the ICU.

BOX 1 **A Model Case.**

Robert^2^ was admitted to the ICU with a severe infection. After a week of critical illness, he became convinced that criminals had captured him. He believed that people around him were torturing him by restraining him (physical restraints) and stabbing him with knives and needles (when changing wound dressings and inserting lines and tubes). He frequently looked towards the window and wondered how to escape. Staff observed a confused, restless, and anxious patient who refused to open his mouth for oral care, resisted being turned in bed, and attempted to hit, kick, or pinch staff during bed washes. Robert often tried to pull out his endotracheal tube and successfully removed the nasogastric tube multiple times. At times, Robert was so stressed that his blood pressure, heart rate and respiratory rate increased to dangerous levels. Weaning him from the mechanical ventilator was complicated by the need to continuously increase sedation to keep Robert and the staff safe. Keeping the wound clean was challenging as Robert constantly moved around in the bed, touching unsanitary sites and removing dressings. Over several days, the staff became exhausted from the continuous efforts to prevent Robert from harming himself or others.

This model case illustrates the multifaceted nature of patient agitation in the ICU. It encompasses all defined attributes of agitation, including excessive motor activity, emotional tension, cognitive impairment, disruption of care, aggression and changes in vital signs. Through this case, the challenges of managing patient agitation are highlighted, emphasising the need for comprehensive and tailored interventions to ensure patient and staff safety.

#### 4.3.2 Related, Contrary, and Illegitimate Cases

Walker and Avant (2019) suggest identifying related, contrary cases or illegitimate cases to get a better picture of what the concept is and what it is not. The case in Box 2 is that of a related case. A related case includes some but not all the attributes.

BOX 2 **Related Case.**

Mrs. Odell^3^ was involved in a traffic accident while riding her push bike to work. The accident resulted in an intracranial bleed. After 4 weeks in the ICU and being weaned from the mechanical ventilator, she exhibited difficulties following conversations and became increasingly withdrawn and anxious, displaying apathy and lethargy. During a visit from a family member, the patient expressed beliefs that foreign forces had taken over, and she begged to be taken home. Following this episode, the patient was diagnosed with delirium.

Mrs. Odell displays some attributes of agitation, including emotional tension and cognitive impairment. However, she does not display excessive motor activity and, therefore, should not be classified as agitated. Mrs. Odell's delirium diagnosis is likely to be sub‐classifiable as hypoactive delirium. While anxiety and delirium may be related to agitation, this case illustrates how they are not identical to patient agitation.

A contrary case would not have any identified attributes of agitation. An example would be a calm and cooperative patient who follows instructions when a nurse asks her to lift her arm to facilitate changing a wound dressing. An illegitimate case is a case where the concept is used incorrectly. For example, a 55‐year‐old patient experiences severe pain following major abdominal surgery. Due to the high acuity and busyness in the ICU, his requests for additional pain relief are not promptly addressed. Consequently, he becomes increasingly frustrated and verbally aggressive, raising his voice and expressing dissatisfaction with the care he is receiving. This patient does not display excessive motor activity and cognitive impairment and thus does not meet the criteria for agitation.

### 4.4 Identifying Antecedents

Antecedents are events that take place before the concept occurs and potentially cause or influence the concept (Walker and Avant 2019). Researchers and experts have tried to understand the antecedents of agitation due to its disruptive and dangerous nature. A comprehensive understanding of factors that lead to and cause behavioural problems can offer valuable information about treatment and prevention. However, identifying the exact events or factors leading to agitation in the ICU can be challenging. For instance, in their study on agitation in the ICU, Jaber et al. (2005) were unable to identify the causes of agitation in one‐third of the patients. Below are the antecedents mentioned in the included literature. The evidence derives from observations and expert opinions, as well as the experiences of patients, families, and clinicians regarding factors or conditions that precede and potentially contribute to agitation. While this section identifies multiple factors that possibly contribute to agitation, it also suggests that the antecedents of agitation may vary among different groups of critically ill patients.

#### 4.4.1 Critical Illness

Various pathophysiological processes associated with critical illness were identified as antecedents to patient agitation. Central Nervous System (CNS) factors include CNS infections (Honiden and Siegel 2010; Cohen et al. 2002), CNS disorders (Mahmood et al. 2018), craniectomy (Chevrolet and Jolliet 2007), insertion of intracranial pressure (ICP) monitors (Mahmood et al. 2018), low Glasgow coma scale (Chen et al. 2014; Burk et al. 2014b; Williamson et al. 2020; Mahmood et al. 2018), seizures (Fraser et al. 2000; Aubanel et al. 2020; Cohen et al. 2002), strokes (Fraser et al. 2000; Wang et al. 2020; Aubanel et al. 2020; Cohen et al. 2002), brain abscesses (Cohen et al. 2002), hepatic encephalopathy (Aubanel et al. 2020), pneumocephalus after craniectomy (Huang et al. 2018), delirium (Almeida et al. 2016; Fraser et al. 2000; Adams et al. 2022; O'Connor et al. 2014; Prendergast et al. 2023; Stewart et al. 2019; Azimaraghi et al. 2023; Aubanel et al. 2020; Honiden and Siegel 2010; Siegel 2003; Cohen et al. 2002; Freeman et al. 2018) and head injury (Fraser et al. 2000; Williamson et al. 2020; Mahmood et al. 2018; Cohen et al. 2002; Freeman et al. 2018). Notably, Almeida et al. (2016) did not find head injury and ICP monitor insertion to be a significant risk factors for postoperative agitation in the ICU.

Circulatory disturbances such as bleeding (Fraser et al. 2000) and hypotension (Fraser et al. 2000; Wang et al. 2020; Whitehouse et al. 2014; Aubanel et al. 2020; Honiden and Siegel 2010; Cohen et al. 2002) may also play a role, along with dysfunction in hepatic, renal, pulmonary, or cardiac systems (Fraser et al. 2000; Burk et al. 2014b; Honiden and Siegel 2010; Cohen et al. 2002). Electrolyte and metabolic abnormalities (Cohen et al. 2002), including low PH (Woods et al. 2004; Burk et al. 2014b; Honiden and Siegel 2010; Freeman et al. 2018), hypoxia (Fraser et al. 2000; Adams et al. 2022; Burk et al. 2014b; Chevrolet and Jolliet 2007; Azimaraghi et al. 2023; Whitehouse et al. 2014; Aubanel et al. 2020; Honiden and Siegel 2010; Cohen et al. 2002; Freeman et al. 2018), hypercapnia (Fraser et al. 2000; Azimaraghi et al. 2023; Whitehouse et al. 2014; Honiden and Siegel 2010), hyperglycaemia (Fraser et al. 2000; Chevrolet and Jolliet 2007; Aubanel et al. 2020; Honiden and Siegel 2010; Cohen et al. 2002), hypoglycaemia (Fraser et al. 2000; Chevrolet and Jolliet 2007; Aubanel et al. 2020; Honiden and Siegel 2010; Cohen et al. 2002), dysnatraemia (Fraser et al. 2000; Whitehouse et al. 2014; Aubanel et al. 2020), uraemia (Cohen et al. 2002) and imbalances in phosphate, calcium, potassium and magnesium (Fraser et al. 2000; Whitehouse et al. 2014; Aubanel et al. 2020) have been described as risk factors for agitation. Elevated levels of heavy metals like lead, mercury, and manganese also contribute to agitation (Cohen et al. 2002).

Additionally, high body temperature (> 38) (Fraser et al. 2000; Jaber et al. 2005; Burk et al. 2014b; Whitehouse et al. 2014; Aubanel et al. 2020; Freeman et al. 2018), nosocomial infections (Adams et al. 2022), pneumonia (Fraser et al. 2000) and sepsis (Fraser et al. 2000; Jaber et al. 2005; Aubanel et al. 2020; Honiden and Siegel 2010; Freeman et al. 2018) are linked to agitation. The severity of illness, indicated by higher Sequential Organ Failure Assessment (SOFA) scores in the respiratory and CNS domains or higher APACHE scores (Jaber et al. 2005; Burk et al. 2014b; Cohen et al. 2002; Freeman et al. 2018), and a medical cause for ICU admission (Jaber et al. 2005; Whitehouse et al. 2014) also correlate with increased patient agitation. Overall, it can be said that a complex interplay of neurological, circulatory, metabolic, infectious, and severity‐related pathophysiological factors is associated with the manifestation of agitation in critically ill patients.

#### 4.4.2 Pharmacological Agents and Other Drugs

Pharmacological agents and other drugs can significantly contribute to agitation in patients, particularly in critical care settings. Key contributors include discontinuation and withdrawal, especially without gradual tapering, from substances such as benzodiazepines (O'Connor et al. 2014), alcohol, opioids, and nicotine (Fraser et al. 2000; Shapira 2002; Whitehouse et al. 2014; Aubanel et al. 2020; Honiden and Siegel 2010; Cohen et al. 2002; Freeman et al. 2018), which can provoke agitation as the body adjusts to the absence of these substances. Sedatives and anxiolytics, such as Lorazepam (Cohen et al. 2002) and propofol (Mahmood et al. 2018), can also cause agitation. Polypharmacy, the use of multiple drugs, often complicates treatment regimes and can lead to agitation due to interactions between antibiotics, muscle relaxants, and various sedatives (Fraser et al. 2000; Cohen et al. 2002). Antipsychotic and psychotropic drugs may also contribute to agitation (Jaber et al. 2005; O'Connor et al. 2014; Azimaraghi et al. 2023; Aubanel et al. 2020; Freeman et al. 2018). Additionally, other agents, such as analgesics (Jaber et al. 2005) anticholinergics (Honiden and Siegel 2010; Cohen et al. 2002), marijuana (Woods et al. 2004), and intoxication from substances (Adams et al. 2022; Honiden and Siegel 2010) can cause or exacerbate agitation. The complexity is further increased by drug side effects (Adams et al. 2022). Benzodiazepines, such as Midazolam, are a good example of drugs that have been reported to cause agitation (Midazolam 2025). Understanding and managing these pharmacological agents and other drugs is crucial for minimising agitation and ensuring effective patient care.

#### 4.4.3 Physical Discomfort and Treatment Related Factors

Physical discomforts can significantly contribute to agitation in critically ill patients. Common sources of discomfort include a full bladder, the need to defecate (Adams et al. 2022; Azimaraghi et al. 2023; Whitehouse et al. 2014; Aubanel et al. 2020; Honiden and Siegel 2010; Cohen et al. 2002), breathing difficulties (Tate et al. 2012; Freeman et al. 2018), and challenges in getting adequate rest or sleep (Malinowski et al. 2020; Shapira 2002; Whitehouse et al. 2014; Honiden and Siegel 2010). Discomfort can also arise from the presence of endotracheal tubes, nasogastric tubes, rectal tubes, intravenous or arterial lines, and urinary catheters (Whitehouse et al. 2014; Honiden and Siegel 2010; Siegel 2003; Cohen et al. 2002; Freeman et al. 2018), as well as from mechanical ventilation (Almeida et al. 2016; Burk et al. 2014b; Aubanel et al. 2020; Cohen et al. 2002).

Additional factors include dry mouth (Honiden and Siegel 2010; Siegel 2003), environmental stimuli such as noise (Malinowski et al. 2020; Prendergast et al. 2023; Shapira 2002; Chevrolet and Jolliet 2007; Cohen et al. 2002) and light (Prendergast et al. 2023; Cohen et al. 2002), nausea (Whitehouse et al. 2014; Honiden and Siegel 2010), and discomfort associated with physical restraints (Adams et al. 2022; Burk et al. 2014b; Freeman et al. 2018). However, Jaber et al. (2005) were unable to confirm that restraints are a risk factor for agitation. Pain from various sources, including procedures, pre‐existing conditions, postoperative pain, suctioning, changing dressings, and repositioning the patient (Almeida et al. 2016; Fraser et al. 2000; Burk et al. 2014b; Prendergast et al. 2023; Shapira 2002; Chevrolet and Jolliet 2007; Azimaraghi et al. 2023; Whitehouse et al. 2014; Aubanel et al. 2020; Siegel 2003; Cohen et al. 2002; Freeman et al. 2018) can also be a significant contributor to agitation, although Huang et al. (2018) did not identify pain as a risk factor for agitation in ICU patients undergoing surgery.

Other discomforts leading to agitation include thirst and hunger (Whitehouse et al. 2014) and uncomfortable body positions (Adams et al. 2022; Tate et al. 2012; Azimaraghi et al. 2023; Honiden and Siegel 2010; Siegel 2003; Cohen et al. 2002). The occurrence and duration of mechanical ventilation (Huang et al. 2018; Malinowski et al. 2020; Freeman et al. 2018), ventilator dyssynchrony (Tate et al. 2012; Whitehouse et al. 2014; Honiden and Siegel 2010; Siegel 2003; Cohen et al. 2002; Freeman et al. 2018) and weaning from mechanical ventilation (Tate et al. 2012) were also factors associated with patient agitation.

#### 4.4.4 Emotional Discomfort

Anxiety (Fraser et al. 2000; Adams et al. 2022; Tate et al. 2012; Malinowski et al. 2020; Shapira 2002; Chevrolet and Jolliet 2007; Azimaraghi et al. 2023; Whitehouse et al. 2014; Honiden and Siegel 2010; Siegel 2003; Cohen et al. 2002; Freeman et al. 2018) and fear (Shapira 2002; Honiden and Siegel 2010; Freeman et al. 2018) are major contributors to agitation in the ICU, together with feelings of being left alone (Tate et al. 2012; Freeman et al. 2018), not being able to communicate (Malinowski et al. 2020; Honiden and Siegel 2010; Cohen et al. 2002) and feeling unfamiliar with the highly technological environment (Shapira 2002). Family interactions can also unintentionally trigger agitation (Tate et al. 2012; Shapira 2002; Freeman et al. 2018).

#### 4.4.5 Patient Specific Characteristics

Patient specific characteristics associated with agitation in the ICU include a history of alcohol and drug abuse (Fraser et al. 2000; Jaber et al. 2005; Burk et al. 2014b; Stewart et al. 2019; Williamson et al. 2020; Whitehouse et al. 2014; Cohen et al. 2002; Freeman et al. 2018), although Huang et al. (2018) did not identify alcohol abuse as a risk factor for postoperative agitation. Patients transferred from another hospital's ICU (Woods et al. 2004) were more likely to experience agitation. Studies also indicated a higher prevalence of agitation in males (Chen et al. 2014; Burk et al. 2014b; Williamson et al. 2020; Cohen et al. 2002), though Jaber et al. (2005) and Huang et al. (2018) found no gender differences.

Coping skills, personality traits (Tate et al. 2012; Shapira 2002), being unmarried (Woods et al. 2004), having a psychiatric history or long‐term use of antidepressants or psychoactive drugs (Burk et al. 2014a, 2014b; Jaber et al. 2005; Chen et al. 2014; Williamson et al. 2020; Whitehouse et al. 2014) are also factors that have been found to contribute to agitation. Weight, age and height are other notable characteristics, with heavier (Burk et al. 2014b), taller (Burk et al. 2014b; Freeman et al. 2018) and younger patients (Woods et al. 2004; Mahmood et al. 2018; Cohen et al. 2002) found to be more prone to agitation, although several researchers (Fraser et al. 2000; Jaber et al. 2005; Huang et al. 2018) found no difference in age, and Wang et al. (2020) found that BMI was not a risk factor.

#### 4.4.6 Uncaring Staff Behaviours

Non‐supportive, inappropriate, and uncaring attitudes from staff can leave patients feeling neglected, isolated, misunderstood, and distressed, which can lead to or exacerbate patient agitation (Tate et al. 2012; Shapira 2002; Freeman et al. 2018).

As evident, agitation is multifactorial, and patients' critical illness and communication difficulties make it difficult to reliably identify the exact causes of agitation. The antecedents include patients' critical illness, including the reason for their ICU admission, pharmacological agents and other drugs, physical and emotional discomfort, the specific characteristics of the patients and uncaring staff behaviours.

### 4.5 Identifying Consequences of Agitation in the ICU

Consequences are the events or incidents that happen as a result of the concept (Walker and Avant 2019). Agitation in the ICU can lead to a multitude of adverse consequences, significantly impacting critically ill patients, their families and healthcare staff. The initial impact of agitation is the interruption of treatment, including the removal of endotracheal tubes (Almeida et al. 2016; Jaber et al. 2005; Chen et al. 2014; Woods et al. 2004; Adams et al. 2022; Burk et al. 2014b; Lucidarme et al. 2010; Stewart et al. 2019; Williamson et al. 2020; Chevrolet and Jolliet 2007; Whitehouse et al. 2014; Aubanel et al. 2020; Mahmood et al. 2018; Freeman et al. 2018) and other devices and interventions such as catheters (e.g., venous, arterial, urinary), nasogastric tubes, wound dressings and physical restraints (Almeida et al. 2016; Jaber et al. 2005; Adams et al. 2022; Burk et al. 2014b; Lucidarme et al. 2010; Williamson et al. 2020; Chevrolet and Jolliet 2007; Aubanel et al. 2020; Freeman et al. 2018).

Agitation also increases the risk of patient falls and injuries (Woods et al. 2004; Adams et al. 2022; Freeman et al. 2022b; Boehm et al. 2021) and leads to increased metabolism, oxygen consumption and myocardial work, precipitating conditions such as ischaemia, arrhythmia, angina (Heily et al. 2023; Chevrolet and Jolliet 2007; Honiden and Siegel 2010) and intracranial hypertension (Chevrolet and Jolliet 2007) leading to haemodynamic and respiratory instabilities (Fraser et al. 2000). Patient agitation often necessitates increased use of sedatives, antipsychotic drugs, opioids, neuromuscular agents, and physical restraints (Fraser et al. 2000; Chen et al. 2014; Woods et al. 2004; Adams et al. 2022; Huang et al. 2018; Lucidarme et al. 2010; Heily et al. 2024, 2023; O'Connor et al. 2014; Shapira 2002; Stewart et al. 2019; Siegel 2003; Freeman et al. 2018). Patient agitation has also been said to cloud the aetiology of underlying diseases, making it challenging to provide timely treatment to the patient (Almeida et al. 2016; Prendergast et al. 2023; Crippen 1999).

Agitation has been linked to prolonged mechanical ventilation (Almeida et al. 2016; Adams et al. 2022; Malinowski et al. 2020; O'Connor et al. 2014; Whitehouse et al. 2014; Freeman et al. 2018; Salazar et al. 2023; Zeller et al. 2017), limited mobility (Adams et al. 2022; Burk et al. 2014b; Whitehouse et al. 2014), skin breakdown from the use of physical restraints (Freeman et al. 2018), an increase in nosocomial infections (Adams et al. 2022; Whitehouse et al. 2014; Aubanel et al. 2020; Freeman et al. 2018) and pneumonia (Mahmood et al. 2018). Agitation has also been associated with an increase in interventions such as diagnostic imaging, surgical procedures, change of dressings and referrals to speciality services (Freeman et al. 2018). Multiple researchers have found that agitation leads to an increased length of stay (Woods et al. 2004; Adams et al. 2022; Prendergast et al. 2023; Stewart et al. 2019; Whitehouse et al. 2014; Aubanel et al. 2020; Honiden and Siegel 2010; Siegel 2003; Freeman et al. 2018), although Mahmood et al. (2018) and Lucidarme et al. (2010) did not find this in their research. Some research suggests that patients experiencing agitation are less likely to be discharged back to their previous living accommodations (Freeman et al. 2018) and may have poorer functional outcomes than patients who were not agitated in the ICU (Huang et al. 2018). Some researchers found an increase in mortality (Almeida et al. 2016; Williamson et al. 2020; Azimaraghi et al. 2023); others (Woods et al. 2004; Lucidarme et al. 2010; O'Connor et al. 2014) found no differences in survival rates between agitated and non‐agitated ICU patients.

Agitation in the ICU impacts patients and their families, leading to patient post‐traumatic stress (Adams et al. 2022; Siegel 2003; Cohen et al. 2002), patient post‐ICU depression (Cohen et al. 2002), and family distress (Adams et al. 2022). The strain on healthcare staff is also considerable. Researchers have described an increased workload (Heily et al. 2024), physical harm to staff (Freeman et al. 2018), moral distress (Freeman et al. 2018), and emotional and physical exhaustion among nurses (Adams et al. 2022; Shapira 2002). These challenges can potentially lead to emotional difficulties, including feelings of guilt, dissatisfaction, and fear, negative attitudes towards patients, less optimal care and increased absenteeism and turnover (Adams et al. 2022).

### 4.6 Defining Empirical Referents

This final step in the concept analysis involves describing how the concept has been measured in the real world. There is currently no gold standard for measuring agitation in ICU. Scales mentioned in the included literature encompass the Riker Sedation Agitation Scale (SAS) (Riker et al. 1999), the Richmond Agitation Sedation Scale (RASS) (Sessler et al. 2002), the Bloomsbury Sedation Scale (Stewart et al. 2019), a modified Ramsay scale (Jaber et al. 2005) and the Motor Activity Assessment Scale (MAAS) (Woods et al. 2004). From these scales, it is clear that agitation occurs on a continuum. However, the various scales lack detailed descriptions, and authors do not agree about what constitutes agitation and often fail to distinguish between anxiety, agitation, aggression, and restlessness. For example, while the Riker Sedation Agitation Scale (SAS) considers anxiety as part of an agitation continuum (Riker et al. 1999) and Burk et al. (2014a) argue that restlessness is a true indication of agitation, the RASS scale suggests that anxiety and restlessness are distinct from agitation (see an overview of different scales in Appendix 4).

## 5 Discussion

Agitation in the ICU is a complex and multifaceted concept that is not well understood. Through this concept analysis, agitation in the ICU can be characterised as excessive motor activity, emotional tension, disruption of care and cognitive impairment, often accompanied by aggression and changes in vital signs. The analysis highlights the need for multifaceted approaches to reducing agitation, as it is influenced by various antecedents, including patient‐specific characteristics, critical illness, and modifiable factors such as physical and emotional discomfort and staff behaviours.

This analysis also highlights the differences between agitation and related concepts such as aggression, anxiety and delirium. Differentiating between these conditions is important for ensuring appropriate and targeted prevention and management. While clinicians often associate agitation with delirium in the ICU (LeBlanc et al. 2018), many delirious patients are hypoactive, exhibiting behaviours like lethargy, withdrawal and minimal engagement with their surroundings (Salazar et al. 2023). These hypoactive behaviours contrast sharply with those of hyperactive, agitated, delirious patients, suggesting that delirium with or without agitation likely requires distinct treatment strategies. Additionally, it is important to acknowledge that agitation often occurs without delirium (Whitehouse et al. 2014). Therefore, recognising agitation as a distinct set of behaviours is essential for appropriate management. Another term that often overlaps with agitated behaviours is anxiety. Anxiety is defined as “a vague uneasy feeling, the source of which is often non‐specific or unknown to the individual” (Mosby 2006, p. 118). Due to the nature of critical illness, anxiety is common in the ICU and can occur with and without agitation (Shdaifat and Al Qadire 2020). Patients can also be agitated without being anxious. Finally, agitation can occur both with and without aggression, but agitated patients who display aggression require particular attention due to the significant risks and challenges they pose (Dazzi et al. 2021). Aggression is often an instinctual behaviour, a response to a perceived threat and overt action intended to cause harm (Dazzi et al. 2021). Cognitive impairment, seen in patients with agitation, can exacerbate this issue, as patients with impaired cognition may misinterpret their environment and perceive non‐threatening situations as threats. This misunderstood perception of threat can trigger aggressive behaviours, making it crucial for clinicians to recognise and address these cognitive challenges early to manage agitation effectively and prevent escalation to aggression (Dazzi et al. 2021).

The variations found in the agitation assessment tools reflect the existing confusion around what constitutes agitation. These variations not only lead to inconsistent evaluation and management of agitation, but also have significant implications for research. When studies use different tools or interpret tools differently, it is challenging to compare results across studies. For example, Burk et al. (2014a); Burk et al. (2014b) saw ‘restlessness’ as part of the agitation picture and therefore defined agitation as a RASS score of +1, while other researchers defined agitation as +2 on the RASS scale (Williamson et al. 2020). Overall, the tools provided very few defining attributes and generally did not include a definition of agitation. While these findings suggest a need for developing a new assessment tool, it is critical to first understand the needs and challenges of clinicians and explore if tools in other areas of healthcare can be useful. Some digital devices claim to measure patient agitation continuously by monitoring physical motion (Saavedra‐Mitjans, Van der Maren, et al. 2024). While the devices may provide useful data, it can be questioned whether they truly measure agitation, as they may not capture other critical aspects such as emotional tension, disruption of care and cognitive impairment.

The existing literature on agitation outside the ICU supports our findings and categorises causes into substances (abuse of drugs, withdrawal), medical conditions (hypoxia, hypotension, head trauma) and psychiatric conditions (personality, mood‐ or psychotic disorders) (Zeller et al. 2017). Pathophysiologically, agitation involves the central nervous system (CNS) with conscious behaviours linked to the cortex, unconscious behaviours to the subcortex and limbic system, and hyperactivity to the basal ganglia‐globus pallidus‐substantia nigra circuit (Simpson 2017). Disruptions in these areas, particularly those related to the production or transport of neurotransmitters, hormones and inflammatory markers, can lead to agitation (Simpson 2017). Social theories, like the reduced stress threshold model developed by Cohen‐Mansfield (2000), suggest that some vulnerable patients, in particular those with lowered coping skills and comprehension, struggle to process and respond to environmental stimuli. When stimuli exceed a person's ability to process the stimuli, there is a risk the person will perceive their environment as stressful, resulting in inappropriate behaviours and agitation. According to this model, it is essential to adjust a patient's environment to their level of capacity and stimuli. Another model, the unmet needs model, suggests that agitation arises from unmet needs, often due to a person's inability to meaningfully express these (Cohen‐Mansfield 2000; Algase et al. 1996). The needs can be physical (pain, fever, sleep disturbance), mental (loneliness) or related to inadequate levels of stimulation. Based on these different theories and the antecedents described in this concept analysis, it is clear that agitation is a reaction to various factors. It is, therefore, critical to identify the causes of agitation and a patient's needs and adjust levels of stimulation before administering medication.

### 5.1 Strengths and Limitations of the Research

This concept analysis offers several important strengths. The search, while not exhaustive, was comprehensive in scope, incorporating primary and secondary, qualitative and quantitative research as well as expert opinion. By including a range of evidence types, this analysis provides a holistic understanding of the concept of agitation in ICUs. According to Risjord (2009), integrating diverse types of evidence helps create a more comprehensive picture of a concept relevant to nursing. Given the limited availability of quantitative research on agitation in the ICU, incorporating the experiences and insights of patients, families, and expert clinicians was crucial for outlining the antecedents and consequences of agitation.

There are also several important limitations to this study that must be acknowledged. The literature search was not exhaustive, as it was limited to three databases and did not include grey literature. Furthermore, the analysis does not critically appraise the quality of the studies included. Concept analysis focuses on how a term has been described in the literature, and while some sources may not meet high methodological standards, they still contribute valuable insights. It is also important to acknowledge that all data, primary and secondary data, was extracted from the papers. This means that while some findings may appear to be supported by a number of studies, it is possible that many of these studies reference the same primary research. Careful interpretation of the findings is required to avoid an overestimation of the breadth of evidence to support individual findings.

Despite these limitations, it is hoped that this analysis will contribute to the development of targeted interventions for clinicians, such as identifying early stages of agitation and preventing its escalation. As the field evolves, this concept analysis may be updated and refined to better inform clinical practice and research in the future.

## 6 Conclusion

This concept analysis aimed to clarify the nature of agitation in the ICU and provide clinicians and researchers with an enhanced understanding of what patient agitation is, what it is caused by, and what consequences it has if not prevented or managed well. This information is essential in understanding how agitation can be better managed. This concept analysis suggests that agitation impacts patients, families, and healthcare professionals. The management of agitation requires a nuanced and multifaceted approach that should focus on patients' critical illness, but also on modifiable components related to physical and emotional discomfort, staff behaviours and the patient's mental health history. Our understanding of patient agitation in the ICU is still limited, and further research into the nuances of agitation and how these can be accurately measured is needed for early detection, tailored interventions, and to determine the effect of such interventions.

## Author Contributions

**Anne Mette N. Adams:** conceptualisation, methodology, validation, investigation, formal analysis, writing the original draft, writing and review and editing. **Diane Chamberlain:** conceptualisation, methodology, data extraction and analysis, validation, writing and review and editing. **Charlotte Brun Thorup:** conceptualisation, data extraction and analysis, writing and review and editing. **Matthew J. Maiden:** data extraction and analysis, writing and review and editing. **Cherie Waite:** data extraction and analysis, writing and review and editing. **Hila Ariela Dafny:** data extraction and analysis, validation, writing and review and editing. **Kay Bruce:** review and editing. **Tiffany Conroy:** conceptualisation, methodology, data extraction and analysis, validation, writing and review and editing.

## Conflicts of Interest

The authors declare no conflicts of interest.

## Supporting information


Appendix S1.

## Data Availability

The data that support the findings of this study are available from the corresponding author upon reasonable request.

## Endnotes

^1^
Definitions were defined as clear and definite statements determining the meaning of a word (Merriam‐Webster n.d.).

^2^
The case and name presented in this article are entirely fictional and have been created for illustrative purposes only.

^3^
The case and name presented in this article are entirely fictional and have been created for illustrative purposes only.

## References

[jan17000-bib-0001] Adams, A. M. N. , D. Chamberlain , M. Grønkjær , C. B. Thorup , and T. Conroy . 2022. “Caring for Patients Displaying Agitated Behaviours in the Intensive Care Unit–A Mixed‐Methods Systematic Review.” Australian Critical Care 35, no. 4: 454–465. 34373173 10.1016/j.aucc.2021.05.011

[jan17000-bib-0002] Adams, A. M. N. , D. Chamberlain , M. Grønkjær , C. B. Thorup , and T. Conroy . 2023. “Nonpharmacological Interventions for Agitation in the Adult Intensive Care Unit: A Systematic Review.” Australian Critical Care 36, no. 3: 385–400. 35513998 10.1016/j.aucc.2022.02.005

[jan17000-bib-0003] Al Ghassani, A. , M. Rababa , and A. Abu Khait . 2021. “Agitation in People With Dementia: A Concept Analysis.” Nursing Forum 56, no. 4: 1015. 34227111 10.1111/nuf.12629

[jan17000-bib-0004] Algase, D. L. , C. Beck , A. Kolanowski , et al. 1996. “Need‐Driven Dementia‐Compromised Behavior: An Alternative View of Disruptive Behavior.” American Journal of Alzheimer's Disease 11, no. 6: 10–19.

[jan17000-bib-0005] Almeida, T. M. , L. C. Azevedo , P. M. Nose , F. G. Freitas , and F. R. Machado . 2016. “Risk Factors for Agitation in Critically Ill Patients.” Rev Bras Ter Intensiva 28, no. 4: 413–419. 28099638 10.5935/0103-507X.20160074 PMC5225916

[jan17000-bib-0006] Aubanel, S. , F. Bruiset , C. Chapuis , G. ChanqueS , and J.‐F. Payen . 2020. “Therapeutic Options for Agitation in the Intensive Care Unit.” Anaesthesia Critical Care & Pain Medicine 39: 639–646. 10.1016/j.accpm.2020.01.009 32777434

[jan17000-bib-0007] Azimaraghi, O. , V. Smith , W. J. Sauer , J. E. Alpert , and M. Eikermann . 2023. “Agitated Patients in the Intensive Care Unit: Guidelines for Causal Rather Than Symptomatic Treatment Are Warranted.” Journal of Intensive Care Medicine 38, no. 2: 238–240. 36373702 10.1177/08850666221138234

[jan17000-bib-0008] Beecher, C. , D. Devane , M. White , R. Greene , and M. Dowling . 2019. “Concept Development in Nursing and Midwifery: An Overview of Methodological Approaches.” International Journal of Nursing Practice 25, no. 1: e12702. 30338594 10.1111/ijn.12702

[jan17000-bib-0009] Boehm, L. M. , A. C. Jones , A. A. Selim , et al. 2021. “Delirium‐Related Distress in the ICU: A Qualitative Meta‐Synthesis of Patient and Family Perspectives and Experiences.” International Journal of Nursing Studies 122: 104030. 10.1016/j.ijnurstu.2021.104030. 34343884 PMC8440491

[jan17000-bib-0010] Burk, R. S. , M. J. Grap , C. L. Munro , C. M. Schubert , and C. N. Sessler . 2014a. “Agitation Onset, Frequency, and Associated Temporal Factors in Critically Ill Adults.” American Journal of Critical Care 23, no. 4: 296–304. 24986170 10.4037/ajcc2014186 PMC4451814

[jan17000-bib-0011] Burk, R. S. , M. J. Grap , C. L. Munro , C. M. Schubert , and C. N. Sessler . 2014b. “Predictors of Agitation in Critically Ill Adults.” American Journal of Critical Care 23, no. 5: 414–423. 25179037 10.4037/ajcc2014714 PMC4451811

[jan17000-bib-0012] Chen, L. , M. Xu , G.‐Y. Li , W.‐X. Cai , and J.‐X. Zhou . 2014. “Incidence, Risk Factors and Consequences of Emergence Agitation in Adult Patients After Elective Craniotomy for Brain Tumor: A Prospective Cohort Study.” PLoS One 9, no. 12: e114239. 25493435 10.1371/journal.pone.0114239 PMC4262354

[jan17000-bib-0013] Chevrolet, J. C. , and P. Jolliet . 2007. “Clinical Review: Agitation and Delirium in the Critically Ill–Significance and Management.” Critical Care 11, no. 3: 214. 17521456 10.1186/cc5787 PMC2206395

[jan17000-bib-0014] Cohen, I. L. , T. J. Gallagher , A. S. Pohlman , J. F. Dasta , E. Abraham , and P. J. Papadokos . 2002. “Management of the Agitated Intensive Care Unit Patient.” Critical Care Medicine 30, no. 1: S97–S123. 10.1097/00003246-200201002-00001 11839894

[jan17000-bib-0015] Cohen‐Mansfield, J. 2000. “Theoretical Frameworks for Behavioral Problems in Dementia.” Alzheimer's Care Today 1, no. 4: 8–21.

[jan17000-bib-0016] Crippen, D. 1999. “Agitation in the ICU: Part One Anatomical and Physiologic Basis for the Agitated State.” Critical Care 3, no. 3: R35–R46. 11094481 10.1186/cc348 PMC137231

[jan17000-bib-0017] Dazzi, F. , M. Valentini , and L. Tarsitani . 2021. “Psychomotor Agitation and Aggression.” In Empathy, Normalization and De‐Escalation: Management of the Agitated Patient in Emergency and Critical Situations, 1–23. Springer.

[jan17000-bib-0018] Devlin, J. W. , Y. Skrobik , C. Gélinas , et al. 2018. “Clinical Practice Guidelines for the Prevention and Management of Pain, Agitation/Sedation, Delirium, Immobility, and Sleep Disruption in Adult Patients in the ICU.” Critical Care Medicine 46, no. 9: e825–e873. 30113379 10.1097/CCM.0000000000003299

[jan17000-bib-0019] Doha, N. M. , T. A. El‐Henawy , and M. M. Mohammed . 2020. “Analgesia and Sedation for Patients in the Intensive Care Unit: A Systematic Review.” Menoufia Medical Journal 33, no. 2: 339.

[jan17000-bib-0020] Fischer, T. , M. Agar , A. Hosie , and A. Teodorczuk . 2020. Unpacking Agitation in Practice: A Call for Greater Precision, 725–726. Oxford University Press. 10.1093/ageing/afaa110 32706853

[jan17000-bib-0021] Fraser, G. L. , B. S. Prato , R. R. Riker , D. Berthiaume , and M. L. Wilkins . 2000. “Frequency, Severity, and Treatment of Agitation in Young Versus Elderly Patients in the ICU.” Pharmacotherapy: The Journal of Human Pharmacology and Drug Therapy 20, no. 1: 75–82. 10.1592/phco.20.1.75.34663 10641977

[jan17000-bib-0022] Freeman, S. , J. Yorke , and P. Dark . 2018. “Patient Agitation and Its Management in Adult Critical Care: A Integrative Review and Narrative Synthesis.” Journal of Clinical Nursing 27, no. 7–8: e1284‐e308. 29314320 10.1111/jocn.14258

[jan17000-bib-0023] Freeman, S. , J. Yorke , and P. Dark . 2022a. “Critically Ill Patients' Experience of Agitation: A Qualitative Meta‐Synthesis.” Nursing in Critical Care 27, no. 1: 91–105. 33949059 10.1111/nicc.12643

[jan17000-bib-0024] Freeman, S. , J. Yorke , and P. Dark . 2022b. “The Patient and Their Family's Perspectives on Agitation and Its Management in Adult Critical Care: A Qualitative Study.” Intensive & Critical Care Nursing 69: 103163. 34893394 10.1016/j.iccn.2021.103163

[jan17000-bib-0025] Hansen‐Flaschen, J. 1994. “Improving Patient Tolerance of Mechanical Ventilation: Challenges Ahead.” Critical Care Clinics 10, no. 4: 659–671. 8000919

[jan17000-bib-0026] Heily, M. , M. Gerdtz , R. Jarden , J. Darvall , and R. Bellomo . 2023. “Anaesthetic Emergence Agitation After Cardiac Surgery: An Intensive Care Staff Survey.” Australian Critical Care 36, no. 5: 832–836. 37616085 10.1016/j.aucc.2022.08.081

[jan17000-bib-0027] Heily, M. , M. Gerdtz , R. J. Jarden , et al. 2024. “Agitation During Anaesthetic Emergence: An Observational Study of Adult Cardiac Surgery Patients in Two Australian Intensive Care Units.” Australian Critical Care 37, no. 1: 67–73. 10.1016/j.aucc.2023.09.003. 37919133

[jan17000-bib-0028] Honiden, S. , and M. D. Siegel . 2010. “Analytic Reviews: Managing the Agitated Patient in the ICU: Sedation, Analgesia, and Neuromuscular Blockade.” Journal of Intensive Care Medicine 25, no. 4: 187–204. 20663774 10.1177/0885066610366923

[jan17000-bib-0029] Huang, H.‐W. , L.‐M. Yan , Y.‐L. Yang , et al. 2018. “Bi‐Frontal Pneumocephalus Is an Independent Risk Factor for Early Postoperative Agitation in Adult Patients Admitted to Intensive Care Unit After Elective Craniotomy for Brain Tumor: A Prospective Cohort Study.” PLoS One 13, no. 7: e0201064. 30024979 10.1371/journal.pone.0201064 PMC6053234

[jan17000-bib-0030] Jaber, S. , G. Chanques , C. Altairac , et al. 2005. “A Prospective Study of Agitation in a Medical‐Surgical ICU: Incidence, Risk Factors, and Outcomes.” Chest 128, no. 4: 2749–2757. 16236951 10.1378/chest.128.4.2749

[jan17000-bib-0031] LeBlanc, A. , F. F. Bourbonnais , D. Harrison , and K. Tousignant . 2018. “The Experience of Intensive Care Nurses Caring for Patients With Delirium: A Phenomenological Study.” Intensive & Critical Care Nursing 44: 92–98. 28993046 10.1016/j.iccn.2017.09.002

[jan17000-bib-0032] Lucidarme, O. , A. Seguin , C. Daubin , et al. 2010. “Nicotine Withdrawal and Agitation in Ventilated Critically Ill Patients.” Critical Care 14, no. 2: R58. 20380688 10.1186/cc8954 PMC2887179

[jan17000-bib-0033] Mahmood, S. , O. Mahmood , A. El‐Menyar , M. Asim , and H. Al‐Thani . 2018. “Predisposing Factors, Clinical Assessment, Management and Outcomes of Agitation in the Trauma Intensive Care Unit.” World Journal of Emergency Medicine 9, no. 2: 105–112. 29576822 10.5847/wjem.j.1920-8642.2018.02.004 PMC5847495

[jan17000-bib-0034] Malinowski, A. , N. J. Benedict , H. Meng‐Ni , L. Kirisci , and S. L. Kane‐Gill . 2020. “Patient‐Reported Outcomes Associated With Sedation and Agitation Intensity in the Critically Ill.” American Journal of Critical Care 29, no. 2: 140–144. 32114616 10.4037/ajcc2020592

[jan17000-bib-0035] Merriam‐Webster . n.d. “Merriam‐Webster Dictionary.” https://www.merriam‐webster.com/dictionary/definition.

[jan17000-bib-0036] Midazolam . 2025. “MIMS Australia.” https://app.emimselite.com/medicineview?id=47ac65b6‐3e02‐4cbe‐ac71‐a5bf01025382&type=abbpi.

[jan17000-bib-0037] Mosby, A. 2006. Mosby's Dictionary of Medicine, Nursing & Health Professions. Mosby. 10.7748/ns2006.02.20.22.36.b426 27319331

[jan17000-bib-0038] O'Connor, H. , N. S. Al‐Qadheeb , A. C. White , V. Thaker , and J. W. Devlin . 2014. “Agitation During Prolonged Mechanical Ventilation at a Long‐Term Acute Care Hospital: Risk Factors, Treatments, and Outcomes.” Journal of Intensive Care Medicine 29, no. 4: 218–224. 23753245 10.1177/0885066613486738

[jan17000-bib-0039] Page, M. J. , J. E. McKenzie , P. M. Bossuyt , et al. 2021. “Updating Guidance for Reporting Systematic Reviews: Development of the PRISMA 2020 Statement.” Journal of Clinical Epidemiology 134: 103–112. 10.1016/j.jclinepi.2021.02.003. 33577987

[jan17000-bib-0040] Prendergast, N. T. , C. A. Onyemekwu , K. M. Potter , P. J. Tiberio , A. E. Turnbull , and T. D. Girard . 2023. “Agitation Is a Common Barrier to Recovery of ICU Patients.” Journal of Intensive Care Medicine 38, no. 2: 208–214. 36300248 10.1177/08850666221134262 PMC10443676

[jan17000-bib-0041] Riker, R. R. , G. L. Fraser , L. E. Simmons , and M. L. Wilkins . 2001. “Validating the Sedation‐Agitation Scale With the Bispectral Index and Visual Analog Scale in Adult ICU Patients After Cardiac Surgery.” Intensive Care Medicine 27, no. 5: 853–858. 11430541 10.1007/s001340100912

[jan17000-bib-0042] Riker, R. R. , J. T. Picard , and G. L. Fraser . 1999. “Prospective Evaluation of the Sedation‐Agitation Scale for Adult Critically Ill Patients.” Critical Care Medicine 27, no. 7: 1325–1329. 10446827 10.1097/00003246-199907000-00022

[jan17000-bib-0043] Risjord, M. 2009. “Rethinking Concept Analysis.” Journal of Advanced Nursing 65, no. 3: 684–691. 19222666 10.1111/j.1365-2648.2008.04903.x

[jan17000-bib-0044] Saavedra‐Mitjans, M. , A. J. Frenette , V. A. McCredie , et al. 2024. “Physicians' Beliefs and Perceived Importance of Traumatic Brain Injury‐Associated Agitation in Critically Ill Patients: A Survey of Canadian Intensivists.” Canadian Journal of Anesthesia/Journal Canadien D'anesthésie 71, no. 2: 264–273. 10.1007/s12630-023-02666-1. 38129356

[jan17000-bib-0045] Saavedra‐Mitjans, M. , S. Van der Maren , N. Gosselin , et al. 2024. “Use of Actigraphy for Monitoring Agitation and Rest‐Activity Cycles in Patients With Acute Traumatic Brain Injury in the ICU.” Brain Injury 1‐7: 692–698. 10.1080/02699052.2024.2341323 38635547

[jan17000-bib-0046] Salazar, W. B. P. , C. M. G. Israel , V. V. R. Antonio , S. S. F. Sofía , and I. Q. R. Austreberto . 2023. “Hyperactive and Hypoactive Delirium: What We Know So Far.” International Journal of Medical Science and Clinical Research Studies 3, no. 7: 1246–1249.

[jan17000-bib-0047] Sano, M. , J. Cummings , S. Auer , et al. 2023. “Agitation in Cognitive Disorders: Progress in the International Psychogeriatric Association Consensus Clinical and Research Definition.” International Psychogeriatrics 2023: 1–13. 10.1017/S1041610222001041 PMC10684256 36880250

[jan17000-bib-0048] Sessler, C. N. , M. S. Gosnell , M. J. Grap , et al. 2002. “The Richmond Agitation‐Sedation Scale: Validity and Reliability in Adult Intensive Care Unit Patients.” American Journal of Respiratory and Critical Care Medicine 166, no. 10: 1338–2002. 12421743 10.1164/rccm.2107138

[jan17000-bib-0049] Shapira, J. 2002. Managing Emotions: Reasoned Detachment Among Nurses in One Surgical Intensive Care Unit. University of California, Los Angeles.

[jan17000-bib-0050] Shdaifat, S. A. , and M. Al Qadire . 2020. “Anxiety and Depression Among Patients Admitted to Intensive Care.” Nursing in Critical Care 27: 106–112. 32844542 10.1111/nicc.12536

[jan17000-bib-0051] Siegel, M. D. 2003. “Management of Agitation in the Intensive Care Unit.” Clinics in Chest Medicine 24, no. 4: 713–725. 14710699 10.1016/s0272-5231(03)00104-7

[jan17000-bib-0052] Simpson, S. 2017. “The Biology of Agitation.” In The Diagnosis and Management of Agitation, 9–20. Cambridge University Press.

[jan17000-bib-0053] Stewart, D. , J. Kinsella , J. McPeake , T. Quasim , and A. Puxty . 2019. “The Influence of Alcohol Abuse on Agitation, Delirium and Sedative Requirements of Patients Admitted to a General Intensive Care Unit.” Journal of the Intensive Care Society 20, no. 3: 208–215. 31447913 10.1177/1751143718787748 PMC6693117

[jan17000-bib-0054] Tate, J. A. , A. Devito Dabbs , L. A. Hoffman , E. Milbrandt , and M. B. Happ . 2012. “Anxiety and Agitation in Mechanically Ventilated Patients.” Qualitative Health Research 22, no. 2: 157–173. 21908706 10.1177/1049732311421616 PMC3598123

[jan17000-bib-0055] Walker, L. O. , and K. C. Avant . 2019. Strategies for Theory Construction in Nursing. 6th ed. Pearson/Prentice Hall.

[jan17000-bib-0056] Wang, H. , D. Hou , X. Tian , et al. 2020. “Risk Factors for Agitation and Hyperactive Delirium in Adult Postcardiotomy Patients With Extracorporeal Membrane Oxygenation Support: An Observational Study.” Perfusion 35, no. 6: 0267659120937549. 10.1177/0267659120937549. 32627668

[jan17000-bib-0057] Whitehouse, T. , C. Snelson , M. Grounds , et al. 2014. “Intensive Care Society Review of Best Practice for Analgesia and Sedation in the Critical Care.”Intensive Care Society, London.

[jan17000-bib-0058] Williamson, D. R. , S. I. Cherifa , A. J. Frenette , et al. 2020. “Agitation, Confusion, and Aggression in Critically Ill Traumatic Brain Injury‐A Pilot Cohort Study (ACACIA‐PILOT).” Pilot and Feasibility Studies 6: 1–10. 33308318 10.1186/s40814-020-00736-5 PMC7729148

[jan17000-bib-0059] Woods, J. C. , L. C. Mion , J. T. Connor , et al. 2004. “Severe Agitation Among Ventilated Medical Intensive Care Unit Patients: Frequency, Characteristics and Outcomes.” Intensive Care Medicine 30, no. 6: 1066–1072. 14966671 10.1007/s00134-004-2193-9

[jan17000-bib-0060] Zeller, S. L. , K. D. Nordstrom , and M. P. Wilson . 2017. The Diagnosis and Management of Agitation. Cambridge University Press.

