# Supplementary material for: Patient Agitation in the Intensive Care Unit: A Concept Analysis
Source: J Adv Nurs. 2025 Apr 25;82(2):1737–49. doi: 10.1111/jan.17000 (PMC12810607; doi:10.1111/jan.17000)
Supplement: Supplementary file 1 — Appendix S1. [file JAN-82-1737-s001.docx]

# Appendix 1

# Overview of search strategies from all databases

Updated searches Nov 2023

Table 1 Medline

| # | **Medline** |
| --- | --- |
| 1 | Critical Illness/ or Critical Care/ or Intensive Care Units/ or Intensive Care/ or Respiration, Artificial/ |
| 2 | (ICU* or ((intensive or critical) adj3 (care or unit*))).tw,kw. |
| 3 | ((critical* adj3 ill*) or ((mechanical* or artificial) adj3 (respiration or ventilat*))).tw,kw. |
| 4 | Or/1-3 |
| 5 | Psychomotor Agitation/ |
| 6 | ("Hyperactive delirium" or agitat* or psychomotor).tw,kw. |
| 7 | Or/5-6 |
| 8 | 4 and 7 |
| 9 | Intensive Care, Neonatal/ or Intensive Care Units, Pediatric/ |
| 10 | (neonatal or p?ediatric).ti,kw. |
| 11 | 9 or 10 |
| 12 | 8 not 11 |
| 13 | limit 15 to english language |

/: MeSH

.tw.kf: Title or abstract, word in author provided keyword

Table 2 CINAHL

| # | **CINAHL for EBSCO** |
| --- | --- |
| S1 | (MH "Critical Illness") OR (MH "Critical Care") OR (MH "Intensive Care Units+") OR (MH "Respiration, Artificial+") |
| S2 | TI ( (ICU* OR ((intensive OR critical) N2 (care OR unit*))) ) OR AB ( (ICU* OR ((intensive OR critical) N2 (care OR unit*))) ) |
| S3 | TI ( ((critical* N2 ill*) OR ((mechanical* OR artificial) N2 (respiration OR ventilat*))) ) OR AB ( ((critical* N2 ill*) OR ((mechanical* OR artificial) N2 (respiration OR ventilat*))) ) |
| S4 | S1 OR S2 OR S3 |
| S5 | (MH "Psychomotor Agitation") OR (MH "Agitation") |
| S6 | TI ( ("Hyperactive delirium" OR agitat* OR psychomotor) ) OR AB ( ("Hyperactive delirium" OR agitat* OR psychomotor) ) |
| S7 | S5 OR S6 |
| S8 | S4 AND S7 |
| S9 | (MH "Intensive Care Units, Pediatric") OR (MH "Intensive Care Units, Neonatal") |
| S10 | TI ( (neonatal OR p#ediatric) ) OR SU ( (neonatal OR p#ediatric) ) |
| S11 | S9 OR s10 |
| S12 | S8 NOT S11 |
| S13 | S11 NOT S14 (narrow by language: English) |

TS: title, abstract, author keywords, and keywords Plus

Table 3 PsycINFO

| # | **PsycINFO** |
| --- | --- |
| 1 | intensive care/ or artificial respiration/ |
| 2 | (ICU* or ((intensive or critical) adj3 (care or unit*))).tw,id. |
| 3 | ((critical* adj3 ill*) or ((mechanical* or artificial) adj3 (respiration or ventilat*))).tw,id. |
| 4 | Or/1-3 |
| 5 | agitation/ |
| 6 | ("Hyperactive delirium" or agitat* or psychomotor).tw,id. |
| 7 | Or/5-6 |
| 8 | 4 and 7 |
| 9 | Intensive Care, Neonatal/ or Intensive Care Units, Pediatric/ |
| 10 | (neonatal or p?ediatric).ti,id. |
| 11 | Or/9-10 |
| 12 | 8 not 11 |
| 13 | limit 15 to english language |

Appendix 2: original PRISMA

Records identified from:

Medline (n =1961)

CINAHL (n=1129)

PsycINFO (n=225)

Duplicates removed (n = 933)

**Identification**

**Included**

Records excluded

(n = 2317)

Reports not retrieved

(n = 0)

Reports assessed for eligibility

(n = 65)

Full texts excluded due to not fitting the inclusion criteria (n= 29).

Articles were excluded if they related to a different condition, were not in English, related to different context or patient population.

Studies included in review

(n = 36)

**Screening**

**Identification of studies via databases**

Records screened (title and abstracts)

(n = 2382)

Reports sought for retrieval

(n = 65)

Figure 1 PRISMA Original

*From:*  Page MJ, McKenzie JE, Bossuyt PM, Boutron I, Hoffmann TC, Mulrow CD, et al. The PRISMA 2020 statement: an updated guideline for reporting systematic reviews. BMJ 2021;372:n71. doi: 10.1136/bmj.n71

Appendix 3

Table 1: Attributes data extraction

| **Categories/**  **Author** | **Excessive motor activity** | **Emotional tension** | **Resisting and/or interrupting care** | **Cognitive impairment** | **Aggressive behaviours** | **Change of vital signs** |
| --- | --- | --- | --- | --- | --- | --- |
| Adams et al. 2021 | x | x | x | x | x |  |
| Almeida et al. 2016 | x | x |  | x |  |  |
| Aubanel et al. 2020 | x | x | x | x |  |  |
| Azimaraghi et al. 2023 | x | x |  |  |  |  |
| Burk et al. 2014B |  | x |  | x | x |  |
| Chevrolet & Jolliet, 2007 | x | x | x | x |  |  |
| Crippen, 1999 | x | x |  |  |  |  |
| Cohen et al. 2002 | x | x | x | x | x | x |
| Fraser et al. 2000 | x | x | x | x | x |  |
| Freeman et al. 2018 | x | x | x | x | x |  |
| Freeman et al. 2022A | x | x |  | x |  |  |
| Heily et al. 2023 | x | x | x |  |  | x |
| Heily et al. 2024 | x |  | x |  | x | x |
| Honiden & Siegel, 2010 | x | x |  |  |  |  |
| Jaber et al. 2005 | x |  |  |  |  |  |
| Mahmood et al. 2018 | x |  | x | x |  |  |
| O'Connor et al. 2014 | x | x |  |  | x |  |
| Prendergast et al. 2023 | x |  | x |  |  |  |
| Shapira, 2002 | x | x | x | x |  | x |
| Siegel, 2003 | x | x | x | x |  |  |
| Stewart et al. 2019 | x | x | x | x |  | x |
| Tate et al. 2012 | x | x | x |  | x | x |
| Whitehouse et al. 2014 | x | x | x | x |  |  |
| Williamson et al. 2020 | x | x | x | x | x |  |
| MAAS scale (Jaber et al. 2005 and Ramsay et al. 1974) | x |  | x | x | x |  |
| RASS scale (Sessler et al. 2002) | x | x | x |  | x |  |
| SAS scale (Riker et al. 1999) | x | x | x |  | x |  |

**Appendix 4: Scales for agitation in ICU**

Please note the ‘sedation’ parts of the scales have been removed due to focus on agitation.

Table 1 MAAS

| Name of scale/ Rating | 4 Restless and cooperative | 5 Agitated | 6 Dangerously agitated,  uncooperative | Notes |
| --- | --- | --- | --- | --- |
| Motor Activity Assessment Scale **(MAAS)** (1) | No external stimulus is required to elicit movement AND patient is picking at sheets or tubes  OR uncovering self and follows commands | No external stimulus is required to elicit movement AND attempting to sit up OR moves limbs  out of bed AND does not consistently follow commands (e.g., will lie down when asked but  soon reverts back to attempts to sit up or move limbs out of bed) | No external stimulus is required to elicit movement AND patient is pulling at tubes or catheters  OR thrashing side to side OR striking at staff OR trying to climb out of bed AND does not calm  down when asked | Used by Woods et al. (2) Scores must be higher than 4 meaning restlessness is not a part of the agitation picture. |

Table 2 RASS

| Name of scale/ Rating | 1 restless | 2 Agitated | 3 Very agitated | 4 Combative | Notes |
| --- | --- | --- | --- | --- | --- |
| Richmond Agitation Sedation Scale **(RASS)** (3) | Anxious, apprehensive but movements not aggressive or vigorous | Frequent non-purposeful movements, fights ventilator | Pulls at or removes tubes, aggressive | Violent, immediate danger to staff | According to Burk et al (4, 5) agitation is RASS of 1 or more.  Other authors (6-10) define agitation as 2 or more.  Williamson et al. (7) include ‘restlessness’ as a sign of agitated behaviours. |

Table 3 SAS

| Name of scale/ Rating | 5 Agitated | 6 Very agitated | Dangerous agitation | Notes |
| --- | --- | --- | --- | --- |
| Riker Sedation Agitation Scale **(SAS)** (11, 12) | Anxious or mildly agitated, attempting to sit up, calms down to verbal instructions | Does not calm despite frequent verbal reminding of limits, requires physical restraints, biting endotracheal tube | Pulling at endotracheal tube, trying to remove catheters, climbing over bedrail, striking at staff, trashing side-to-side | Used by several authors (13-16). Unclear if anxiety, which is described under ’Agitation’ is a part of the agitation picture. This scale does not mention restlessness as part of the agitation picture |

Table 4 Ramsay and Bloomsbury

| Name of scale/ Rating | Description | Notes |
| --- | --- | --- |
| Ramsay Sedation Scale (17, 18) | Patient is anxious and agitated or restless or both | This scale is not used by any of the included studies |
| Modified Ramsay Sedation Scale (17, 19) | Patient agitated or fidgeting or bucking ventilator despite  attempts of the staff to calm the patient | Used by Jaber et al. (19). Unclear what agitation is. Scale suggests that fidgeting or bucking ventilator may be something different to agitation. |
| Bloomsbury Sedation Scale | Restless or agitated | Stewart et al. (20) define agitation as a score of 3 |

**References:**

1. Devlin JW, Boleski G, Mlynarek M, Nerenz DR, Peterson E, Jankowski M, et al. Motor Activity Assessment Scale: A valid and reliable sedation scale for use with mechanically ventilated patients in an adult surgical intensive care unit. Critical Care Medicine. 1999;27(7):1271-5.

2. Woods JC, Mion LC, Connor JT, Viray F, Jahan L, Huber C, et al. Severe agitation among ventilated medical intensive care unit patients: frequency, characteristics and outcomes. Intensive care medicine. 2004;30(6):1066-72.

3. Sessler CN, Gosnell MS, Grap MJ, Brophy GM, O'Neal PV, Keane KA, et al. The Richmond Agitation-Sedation Scale: Validity and reliability in adult intensive care unit patients. American Journal of Respiratory and Critical Care Medicine. 2002;166(10):1338-2002.

4. Burk RS, Grap MJ, Munro CL, Schubert CM, Sessler CN. Predictors of agitation in critically ill adults. American Journal of Critical Care. 2014;23(5):414-23.

5. Burk RS, Grap MJ, Munro CL, Schubert CM, Sessler CN. Agitation onset, frequency, and associated temporal factors in critically ill adults. Am J Crit Care. 2014;23(4):296-304.

6. Aubanel S, Bruiset F, Chapuis C, ChanqueS G, Payen J-F. Therapeutic options for agitation in the intensive care unit. Anaesthesia Critical Care & Pain Medicine. 2020.

7. Williamson DR, Cherifa SI, Frenette AJ, Saavedra Mitjans M, Charbonney E, Cataford G, et al. Agitation, confusion, and aggression in critically ill traumatic brain injury-a pilot cohort study (ACACIA-PILOT). Pilot and feasibility studies. 2020;6:1-10.

8. Heily M, Gerdtz M, Jarden RJ, Yap CY, Darvall J, Coventry AE, et al. Agitation during anaesthetic emergence: An observational study of adult cardiac surgery patients in two Australian intensive care units. Australian Critical Care. 2024;37(1):67-73.

9. Heily M, Gerdtz M, Jarden R, Darvall J, Bellomo R. Anaesthetic emergence agitation after cardiac surgery: An intensive care staff survey. Australian Critical Care. 2023;36(5):832-6.

10. Almeida TM, Azevedo LC, Nose PM, Freitas FG, Machado FR. Risk factors for agitation in critically ill patients. Rev Bras Ter Intensiva. 2016;28(4):413-9.

11. Riker RR, Picard JT, Fraser GL. Prospective evaluation of the Sedation-Agitation Scale for adult critically ill patients. Critical care medicine. 1999;27(7):1325-9.

12. Ryder-Lewis MC, Nelson KM. Reliability of the Sedation-Agitation Scale between nurses and doctors. Intensive and Critical Care Nursing. 2008;24(4):211-7.

13. Malinowski A, Benedict NJ, Meng-Ni H, Kirisci L, Kane-Gill SL. Patient-reported outcomes associated with sedation and agitation intensity in the critically ill. American Journal of Critical Care. 2020;29(2):140-4.

14. Chen L, Xu M, Li G-Y, Cai W-X, Zhou J-X. Incidence, risk factors and consequences of emergence agitation in adult patients after elective craniotomy for brain tumor: a prospective cohort study. PloS one. 2014;9(12):e114239.

15. Lucidarme O, Seguin A, Daubin C, Ramakers M, Terzi N, Beck P, et al. Nicotine withdrawal and agitation in ventilated critically ill patients. Critical Care. 2010;14(2):R58.

16. Huang H-W, Yan L-M, Yang Y-L, He X, Sun X-M, Wang Y-M, et al. Bi-frontal pneumocephalus is an independent risk factor for early postoperative agitation in adult patients admitted to intensive care unit after elective craniotomy for brain tumor: A prospective cohort study. PloS one. 2018;13(7):e0201064.

17. Ramsay M, Savege T, Simpson B, Goodwin R. Controlled sedation with alphaxalone-alphadolone. Br med J. 1974;2(5920):656-9.

18. Rasheed AM, Amirah MF, Abdallah M, Parameaswari P, Issa M, Alharthy A. Ramsay sedation scale and richmond agitation sedation scale: A Cross-sectional study. Dimensions of Critical Care Nursing. 2019;38(2):90-5.

19. Jaber S, Chanques G, Altairac C, Sebbane M, Vergne C, Perrigault P-F, et al. A prospective study of agitation in a medical-surgical ICU: incidence, risk factors, and outcomes. Chest. 2005;128(4):2749-57.

20. Stewart D, Kinsella J, McPeake J, Quasim T, Puxty A. The influence of alcohol abuse on agitation, delirium and sedative requirements of patients admitted to a general intensive care unit. Journal of the Intensive Care Society. 2019;20(3):208-15.
